# Fertile Tetraploids: New Resources for Future Rice Breeding?

**DOI:** 10.3389/fpls.2020.01231

**Published:** 2020-08-11

**Authors:** Yohei Koide, Daichi Kuniyoshi, Yuji Kishima

**Affiliations:** Laboratory of Plant Breeding, Research Faculty of Agriculture, Hokkaido University, Sapporo, Japan

**Keywords:** rice, tetraploid, fertility, pollen, seed

## Abstract

Ploidy manipulation is an efficient technique for the development of novel phenotypes in plant breeding. However, in rice (*Oryza sativa* L.), severe seed sterility has been considered a barrier preventing cultivation of autotetraploids since the 1930s. Recently, a series of studies identified two fertile autotetraploids, identified herein as the PMeS (Polyploid Meiosis Stability) and Neo-Tetraploid lines. Here, we summarize their characteristics, focusing on the recovery of seed fertility, and discuss potential future directions of study in this area, providing a comprehensive understanding of current progress in the study of fertile tetraploid rice, a classical, but promising, concept for rice breeding.

## Introduction

The phenomenon of polyploidy, which refers to the multiplication of chromosome sets within cells, often doubling a normal (diploid) set into a quadruple (tetraploid) set, is a widespread and distinctive feature of the higher plants (Stebbins, 1950). Polyploids are traditionally classified into autopolyploids, which originate from a single parent species (xx to xxxx), and allopolyploids, which originate from two hybridizing species (xx + yy to xxyy) (Clark and Donghe, 2018). In plants, polyploidization occurs frequently, and 25%–30% of flowering plants are suggested to be current polyploids, which have not yet diploidized (Van de Peer et al., 2017). The adaptive potential of polyploidy has long been discussed. Van de Peer et al. (2017) summarized the adaptive potential of polyploids with respect to the effects of interaction between species (i.e., changes in modes of reproduction and changes in interactions with pollinators and herbivores), environmental robustness (i.e., increasing tolerance to abiotic stresses), and species diversification. These changes in adaptive potential are reported to be caused by changes in the genomes/transcriptomes of the polyploid, including structural changes in genomes/genes, and transcriptional and functional alterations in duplicated genes (for a detailed review of these changes, see Renny-Byfield and Wendel (2014)]. Because the function of a duplicated gene should be redundant at the time of polyploidization, it can accumulate mutations, which may affect its function without reducing the fitness of the individual (Blanc and Wolfe, 2004). It is therefore widely accepted that polyploids have greater mutational robustness than diploids (Van de Peer et al., 2009). Fawcett et al. (2009) showed that the majority of the most recent duplication events in flowering plants are clustered in time and seem to coincide with the period of the most recent mass extinction. This suggests that polyploid plants coped better than diploid plants with the markedly changed environments of that period (Van de Peer et al., 2009).

In crop species, it has been frequently observed that polyploidization causes plants to grow larger, more quickly, and with higher yields, compared to their diploid relatives (Renny-Bayfield and Wendel, 2014), although some studies suggest that polyploidization did not have a selective advantage over diploid genomes during the domestication process (Hilu, 1993). In addition to improved crop yield, recent studies have shown that polyploidy generates novel or diversified characteristics such as changes to grain threshing properties (Zhang et al., 2011) and grain hardness (Chantret et al., 2004) in wheat, flowering time in sunflower (Blackman et al., 2010), growing condition in coffee (Combes et al., 2012), and quality of fiber in cotton (Jiang et al., 1998). These studies prompted us to survey the potential capacity for polyploidization in rice, a staple food for four billion people globally (CGIAR, http://ricecrp.org/), as a means of providing new genetic materials for future crop improvement. In this review, we focus on autotetraploid rice, especially its fertility, because low seed fertility has been regarded as a barrier obstructing its use. In the course of summarizing two pioneering studies on autotetraploid rice with high seed fertility, we will discuss potential future directions for ploidy manipulation in rice, a classical but promising concept.

## History of the Study of Tetraploid Rice

Asian cultivated rice (*Oryza sativa* L.) is a diploid species with two sets of 12 chromosomes (2n = 24). [Although Asian cultivated rice may have experienced whole genome duplication more than 96 million years ago (Guo et al., 2019), this species is regarded as diploid, as it did not experience a “recent” polyploidization. In this review, we therefore regard Asian cultivated rice as a diploid species and tetraploid rice as containing two sets of 24 chromosomes]. As far as we know, no autotetraploid or allotetraploid rice varieties are used in agriculture. The first report of spontaneously autotetraploid rice was by Nakamori (1933), 2 decades after the determination of its chromosome number (Kuwada, 1910). Soon after the 1933 report, Ichijima (1934) reported artificially induced tetraploids. Some subsequent studies concerning the artificial induction of tetraploidy in rice have been reported (Cua, 1950; Oka, 1953). According to He et al. (2010), research on polyploid rice breeding was initiated in 1953 in China.

## Low Seed Fertility: A Barrier Preventing Tetraploid Rice Use

In general, polyploidization confers greater stress tolerance by fostering slower development, delayed reproduction, longer life span, improved defense against pathogens and herbivores, larger seeds, and lower reproductive effort with greater emphasis on vegetative reproduction [Hilu, 1993, see also review by Levin (1983)]. In rice, large grain and leaf size are often observed in autotetraploid plants. In addition, Tu et al. (2014) reported higher salt tolerance in autotetraploid rice than in diploid rice. These studies suggest the potential usefulness of autotetraploid rice for breeding. However, low numbers of spikelets per panicle and low fertility have frequently observed in tetraploid rice throughout its known existence. Oka (1954) surveyed the phenotypes of a diverse set of autotetraploid rice varieties. Although greater vegetative than reproductive growth was not specifically indicated (**Table 1**), he showed that seed fertility in tetraploid varieties ranged from 0% to 55% (Oka, 1954; Oka, 1955). This low seed set rate has been the main barrier preventing use of autotetraploid for rice breeding (Cai et al., 2007).

**Table 1:** General characteristics of tetraploid rice lines (summary of Oka, 1954).

Traits	Characteristics in tetraploids
Panicle length	Increased (0.95 to 1.30)
Grain length	Increased (1.15 to 1.30)
Awn	Developed
Plant height	Reduced (0.65 to 1.00)
Spikelet number per panicle	Reduced (0.50 to 0.90)
Panicle number per plant	Reduced (0.40 to 0.70)
Seed fertility	0% to 55%
Pollen fertility	55% to 95%

Numbers in parenthesis indicate ratio of phenotype value of tetraploids compared to diploids.

Lower polyploid fertilities have been considered to be due to abnormal behavior of the chromosomes during meiosis (Bomblies et al., 2016). In the first division of meiosis, a pair of homologous chromosomes forms a bivalent, which later segregates. However, in polyploids, especially autopolyploids, the proper formation of bivalents is often inhibited, because homology among three or more chromosomes means that they fail to provide the special intrinsic cues necessary for normal, diploid-like segregation (Bomblies et al., 2016). In autotetraploid rice, chromosomal behavior at meiosis has been surveyed by Cai et al. (2007) and Wu et al. (2014). Cai et al. (2007) found that a tetraploid line, Dure-4X, displays abnormal meiotic behaviors including a higher rate of multivalents, univalents, and trivalents during prophase, lagging chromosomes during metaphase, and micronuclei during anaphase and telophase. In addition to such abnormalities, asynchrony of chromosomes and abnormal cell shape are also observed in meiosis in pollen mother cells in tetraploids with low fertility (Wu et al., 2014). These studies suggested that abnormal chromosomal behavior is one of the reasons for low seed fertility in autotetraploid rice. Recent studies have revealed the differences of epigenetic state and transcriptomes between the diploid and tetraploid rice (Xu et al., 2014; Zhang et al., 2015; Li et al., 2018). Zhang et al. (2015) suggested that the increased methylation level of transposable elements (TE) alters the expression of genes near by the TE in autotetraploid rice. Transcriptomic differences, including the expression of small RNAs, long non-coding RNAs, meiosis-related genes, and carbohydrate metabolism-related genes have been suggested to be related to meiotic abnormalities and the subsequent reduction of pollen fertility in autotetraploid rice (Li et al., 2016; Li et al., 2017; Chen et al., 2018; Li et al., 2020).

## PMeS (Polyploid Meiosis Stability) and Neo-Tetraploidy: Emerging Fertile Autotetraploid Rice

Although its low seed fertility has long been a barrier preventing the use of autotetraploid rice in breeding, two new autotetraploid rice series with high seed and pollen fertilities, identified herein as the PMeS and Neo-Tetraploid lines, were developed recently (**Table 2**). The PMeS lines have been developed and analyzed by Cai et al. (2007) and subsequent studies (He et al., 2010; He Y. C. et al., 2011; Tu et al., 2014). They derive from the progenies of crosses between *indica* and *japonica* rice subspecies. Unlike other autotetraploid rice lines, PMeS lines do not show abnormal chromosomal behavior at meiosis. In addition, their pollen development pattern appears normal at all stages, lacking the many abnormalities seen in other autotetraploid rice lines (He et al., 2010). These results suggest that stable meiosis, timely tapetum degradation, and normal mitochondrial development were the critical factors ensuring the high pollen fertility of PMeS lines (He et al., 2010). Recently, Xiong et al. (2019) revealed that the rice homolog of the gene *MND1*, which plays a role in meiosis in yeast and *Arabidopsis*, also plays a crucial role in improving the seed set rate in PMeS lines. They showed that the *OsMND1* expression level in panicles of a PMeS line was higher than in the diploid lines or in the other autotetraploid line with low seed set rate and *OsMND1* overexpression improved pollen fertility and seed set. The meiotic behavioral observations suggested that the effects of *OsMND1* might be maintaining the balance of synapsis and recombination (Xiong et al., 2019). However, it is still unknown why the expression level of *OsMND1* was lower in autotetraploid line with low seed set rate than the PMeS line. More research is necessary for a full understanding of the molecular mechanism of normal meiosis in PMeS lines (Xiong et al., 2019).

**Table 2:** Comparison of two fertile tetraploid rice series.

	Neo-Tetraploid lines	PMeS lines
Line name	Huaduo1, 2, 3, 4, 5, 8^a,b,c,d^	Sg99012, HN-2026-4X^e,f^
Cross combination	Inter-subspecific (*japonica* and *indica*)	Inter-subspecific (*japonica* and *indica*)^f^
Seed set	more than 68%^a,b^	Over 80%^e,f^
Pollen fertility	more than 92%^a^	84.15% (stained), 78.52% (germinated)^g^
Meiotic behavior in male gametes	Few abnormality were detected^a^	Normal^e^
Seed set of F1	83.07% (when crossed with *japonica* varieties)^a^	67.18% (when crossed with Ballila-4X)^e^
	76.45% (when crossed with *indica* varieties)^a^	

^a^Bei et al., 2019, ^b^Guo et al., 2017, ^c^Chen et al., 2019, ^d^Ghaleb et al., 2020, ^e^He Y. C. et al., 2011, ^f^Cai et al., 2007, ^g^He et al., 2010.

The other autotetraploid rice varieties with high seed and pollen fertilities were named the Neo-Tetraploid lines by Guo et al. (2017). These lines derived from the progenies of crosses between T44 (96025) and T45 (Jackson-4X) and showed a seed set rate of more than 80%, while their parents (T44 and T45) showed less than 32% (Guo et al., 2017; Bei et al., 2019). Cytological observation of the meiotic stages in Neo-Tetraploid rice lines showed that fewer abnormalities in these lines than their autotetraploid parents, which displayed different chromosomal configurations at diakinesis (Bei et al., 2019). Surprisingly, a total of 324 genes in the Neo-Tetraploid rice genome showed new mutations, which do not exist in its parents’ genomes (Bei et al., 2019). By means of a transcriptome analysis, Bei et al. (2019) suggested that genomic structural reprogramming, DNA variations, and differential expression of some important meiosis- and epigenetics-related genes might be associated with the high fertility of Neo-Tetraploid lines.

## Autotetraploid Rice and Hybrid Sterility

Autotetraploid rice varieties with high seed fertilities are also considered to be important resources for hybrid rice breeding (Guo et al., 2017). Interestingly, both PMeS and Neo-Tetraploid lines produce F1 progenies with high seed setting rates when crossed with other autotetraploid rice lines (**Table 2**, He Y. C. et al., 2011; Guo et al., 2017). In PMeS lines, He Y. C. et al. (2011) showed that the seed set rate of an F1 hybrid of Balilla-4X × HN2026-4X (a PMeS line) was 67.18%, while it was 37.26% in the F1 of Balilla-4X × NJ11-4X (a non-PMeS line). They suggested that the normal meiotic behavior in lines with a PMeS background led to the high frequency of successful fertilization, normal embryo development, and high seed set rate observed in its F1 progeny.

In Neo-Tetraploid lines, the agronomic traits of various autotetraploid hybrids were examined by Guo et al. (2017). They found that the seed setting rate in the hybrids of Neo-Tetraploid rice was significantly higher than in the hybrids generated from other autotetraploid lines. Their transcriptome analysis revealed that genes expressed differently by the hybrid and its parents included meiosis stage-specific- and meiosis-related genes, such as *RAD51* and *SMC2*. Because *RAD51* and *SMC2* are involved in recombination between homologous chromosome and control of chromosomal structure, respectively, during meiosis (Aya et al., 2011), their transcriptional changes should affect to the fertility observed in autotetraploid hybrids. However, the understanding of the cause of transcriptional changes in hybrids still remains a challenging.

The seed fertility of autotetraploid hybrids is also controlled by several hybrid sterility loci. At such loci in rice, several alleles exist; some of them interact in the heterozygous state to cause abortion of gametes (see the review of Koide et al., 2008b). At this locus, the allele that does not cause abortion of gametes is called the neutral allele (Ikehashi and Araki, 1986; Koide et al., 2008a). He et al. (2011b) and Wu et al. (2015) showed that interaction between three hybrid sterility loci (*Sa*, *Sb*, and *Sc*) has significant effects on the pollen fertility of autotetraploid hybrids, and that pollen fertility further decreased with increasing allelic interaction. They also found abnormal chromosomal behavior in the hybrids with low pollen fertility and suggested that the gene interactions of hybrid sterility loci tend to increase the chromosomal abnormalities that cause the partial abortion of male gametes, leading to the decline in seed set of the autotetraploid rice hybrids (He et al., 2011b). Such abnormalities in chromosomal behavior were reduced in hybrids with neutral alleles (*Sa*^n^ and *Sb*^n^) at loci *Sa* and *Sb*, respectively (Wu et al., 2017). Chen et al. (2019) developed hybrids between the Neo-Tetraploid line and autotetraploid varieties with neutral alleles. They found an improvement of seed set rate and suggested that meiosis-related and meiosis-specific genes, and those related to saccharide metabolism and starch synthase, were involved in these heterozygote-specific phenotypes (Chen et al., 2019).

## Discussions and Conclusions

These two series of pioneer studies on the PMeS and Neo-Tetraploid lines have opened the door to the use of autotetraploid varieties in rice breeding and cultivation. To facilitate the development of fertile autotetraploid lines, further research aimed at understanding the origin of the genes/quantitative trait loci (QTLs) responsible for high fertility in these tetraploid rice varieties will be necessary. Both the PMeS and Neo-Tetraploid rice lines have been developed from the progenies of crosses between the *indica* and *japonica* rice subspecies. Since the parental autotetraploid varieties show seed sterility, one possibility for the origin of high-fertility genes/QTLs in the new varieties’ progenies is the nature of interactions between genes (or genomic regions) derived from different autotetraploid parents. If so, a QTL analysis of high seed fertility could be performed by developing recombinant inbred lines derived from crosses between autotetraploids. The Neo-Tetraploid lines show different expression of some important meiosis stage specific- and meiosis-related genes (Guo et al., 2017; Bei et al., 2019). Genes/QTLs responsible for high seed fertility might control the expression of these. Interestingly, the PMeS and Neo-Tetraploid lines show high seed fertility not only when selfing, but also in hybrids with other autotetraploid rice strains (He Y. C. et al., 2011; Guo et al., 2017). These results suggest that the genes/QTLs responsible for high seed fertility in PMeS and Neo-Tetraploid lines act dominantly, suppressing unstable chromosomal behavior during meiosis in hybrids. If this is so, the question arises as to why the initial hybrids of *indica* and *japonica* used for developing the PMeS and Neo-Tetraploid lines did not show high seed fertility. Another possibility for the origin of genes/QTLs responsible for high seed fertility is the new appearance of these genes/QTLs during the line selection process. The finding of new mutations, absent from the parental genome, in a Neo-Tetraploid line (Bei et al., 2019) may support this scenario. The activation of TEs followed by alteration of the epigenetic state (Zhang et al., 2015; Guo et al., 2017; Bei et al., 2019) may cause new appearance of genes/QTLs in tetraploid rice. Further study is necessary to unclear why many such mutations would occur in the autotetraploid lines.

Although their origin is unclear, identifying the genes responsible for high seed fertility in the PMeS and Neo-Tetraploid lines will enable the transfer of this technique to other autotetraploid rice varieties through marker-assisted selection or genome editing. A diverse set of fertile autotetraploid rice varieties will greatly increase their potential usefulness in future agriculture. Ploidy manipulation, a classical concept, will revive in the field of rice breeding in the near future.

## Author Contributions

YKo, DK, and YKi contributed to conceptualization of this review. YKo wrote original draft. DK and YKi reviewed and edited the original manuscript.

## Funding

YKo and YKi are funded by JSPS KAKENHI Grant Numbers 18K05565 and 19H00937, respectively.

## Conflict of Interest

The authors declare that the research was conducted in the absence of any commercial or financial relationships that could be construed as a potential conflict of interest.
